# Gene therapy with AR isoform 2 rescues spinal and bulbar muscular atrophy phenotype by modulating AR transcriptional activity

**DOI:** 10.1126/sciadv.abi6896

**Published:** 2021-08-20

**Authors:** Wooi F. Lim, Mitra Forouhan, Thomas C. Roberts, Jesse Dabney, Ruth Ellerington, Alfina A. Speciale, Raquel Manzano, Maria Lieto, Gavinda Sangha, Subhashis Banerjee, Mariana Conceição, Lara Cravo, Annabelle Biscans, Loïc Roux, Naemeh Pourshafie, Christopher Grunseich, Stephanie Duguez, Anastasia Khvorova, Maria Pennuto, Constanza J. Cortes, Albert R. La Spada, Kenneth H. Fischbeck, Matthew J.A. Wood, Carlo Rinaldi

**Affiliations:** 1 Department of Paediatrics, University of Oxford, Oxford, UK.; 2 Department of Physiology, Anatomy and Genetics, University of Oxford, Oxford, UK.; 3 RNA Therapeutics Institute, University of Massachusetts Medical School, Worcester, MA, USA.; 4 Neurogenetics Branch, National Institute of Neurological Disorders and Stroke (NINDS), Bethesda, MD, USA.; 5 Northern Ireland Centre for Stratified Medicine, Biomedical Sciences Research Institute, Londonderry, UK.; 6 Department of Biomedical Sciences, University of Padova, Padova, Italy.; 7 Venetian Institute of Molecular Medicine (VIMM), Padova, Italy.; 8 Department of Neurology, Duke Center for Neurodegeneration and Neurotherapeutics, Duke University School of Medicine, Durham, NC, USA.; 9 Departments of Pathology and Laboratory Medicine, Neurology, and Biological Chemistry and the UCI Institute for Neurotherapeutics, University of California, Irvine, Irvine, CA, USA.; 10 MDUK Oxford Neuromuscular Centre, University of Oxford, Oxford, UK.

## Abstract

Spinal and bulbar muscular atrophy (SBMA) is an X-linked, adult-onset neuromuscular condition caused by an abnormal polyglutamine (polyQ) tract expansion in androgen receptor (AR) protein. SBMA is a disease with high unmet clinical need. Recent studies have shown that mutant AR-altered transcriptional activity is key to disease pathogenesis. Restoring the transcriptional dysregulation without affecting other AR critical functions holds great promise for the treatment of SBMA and other AR-related conditions; however, how this targeted approach can be achieved and translated into a clinical application remains to be understood. Here, we characterized the role of AR isoform 2, a naturally occurring variant encoding a truncated AR lacking the polyQ-harboring domain, as a regulatory switch of AR genomic functions in androgen-responsive tissues. Delivery of this isoform using a recombinant adeno-associated virus vector type 9 resulted in amelioration of the disease phenotype in SBMA mice by restoring polyQ AR–dysregulated transcriptional activity.

## Teaser

AR isoform 2–encoded variant acts as a transcriptional modulator of AR genomic activity and is a target for therapy for SBMA.

## INTRODUCTION

Spinal and bulbar muscular atrophy (SBMA), or Kennedy’s disease, is an X-linked, adult-onset neuromuscular disease caused by a CAG (cytosine-adenine-guanine) trinucleotide repeat expansion encoding a polyglutamine (polyQ) tract in the androgen receptor (AR) (*1*) and affecting 1 in 50,000 men worldwide (*2*). SBMA is frequently misdiagnosed with other neuromuscular diseases (*3*). In addition, recent findings indicate a significantly larger proportion of individuals among the general population carrying pathological ranges of polyQ disease–associated alleles, including *AR*, than what expected from previous studies (*4*, *5*), overall suggesting that the prevalence of this disease is largely underestimated.

AR is a member of the nuclear receptor superfamily, a class of transcription factors activated by steroid hormones (*6*), and is involved in several essential physiological processes such as cell growth and survival through mechanisms both dependent and independent on DNA binding (*7*). Nuclear receptors share a common three-domain structure, comprising an intrinsically disordered N-terminal domain (NTD), with potent transcriptional activation or repression capability, a central two zinc finger motif DNA binding domain (DBD), and a well-conserved C-terminal ligand-binding domain (LBD), responsible for ligand recognition (*6*). Given their modular structure, expression of isoforms resulting from alternative splicing or starting and termination events has profound effects on nuclear receptor activity and function.

SBMA individuals develop muscle weakness and atrophy as a consequence of lower motor neuron and skeletal muscle degeneration and usually become wheelchair-bound within 15 to 20 years from disease onset (*8*). Toxicity is dependent on the presence of AR ligand testosterone or dihydrotestosterone (DHT), and cell dysfunction and death ultimately result from profound alterations of cellular processes mainly by a gain-of-function mechanism (*9*–*14*). The mutation also leads to a partial loss of AR function, which accounts for certain aspects of the disease, such as the partial androgen insensitivity syndrome frequently observed in patients with SBMA (*15*, *16*). In addition to the proteotoxic stress posed by the mutant protein, the expanded polyQ tract severely affects AR transcriptional activity, with significant alterations of expression of genes containing the androgen-responsive element (ARE) motif (*10*, *13*, *14*).

Previous studies in a *Drosophila* model of SBMA have shown that interaction between the AR and its transcriptional coregulators through the AR activation function 2 (AF2) domain and binding of AR to target genes are both required to start the disease (*17*). A therapeutic approach that is able to selectively modulate polyQ AR transcriptional dysregulation, without interfering with other AR critical functions, holds great promise for the treatment of SBMA and is not currently available. Despite recent advancements of the understanding of disease mechanisms, to this date, SBMA remains a condition with high unmet clinical need. The use of antiandrogens has shown marginal benefits in phase 2 trials (*18*) and no definite amelioration of the motor functions in patients in larger, multicenter randomized-controlled trials (*19*, *20*). AR-silencing strategies have shown encouraging results in preclinical models but are hampered by the risks of long-term, widespread AR loss (*21*). Alternatively, small molecules that modulate AR coregulator–binding affinity are an attractive therapeutic avenue (*22*–*24*). Nevertheless, the lack of full understanding of their mechanisms of action, potential off-target effects, and unfavorable pharmacokinetic profiles limits their clinical applicability.

In our study, we establish a physiological role of one of the naturally occurring AR isoforms, i.e., AR isoform 2 (AR-2), in fine-tuning full-length AR transcriptional activity. Furthermore, by selectively targeting mutant AR transcriptional dysregulation, we provide proof-of-principle evidence for efficacy and safety of a gene therapy strategy based on delivery of this AR variant to treat SBMA, confirming the translational potential of this strategy.

## RESULTS

### AR-2 isoform gene expression profiling in human tissues

**Fig. 1:**
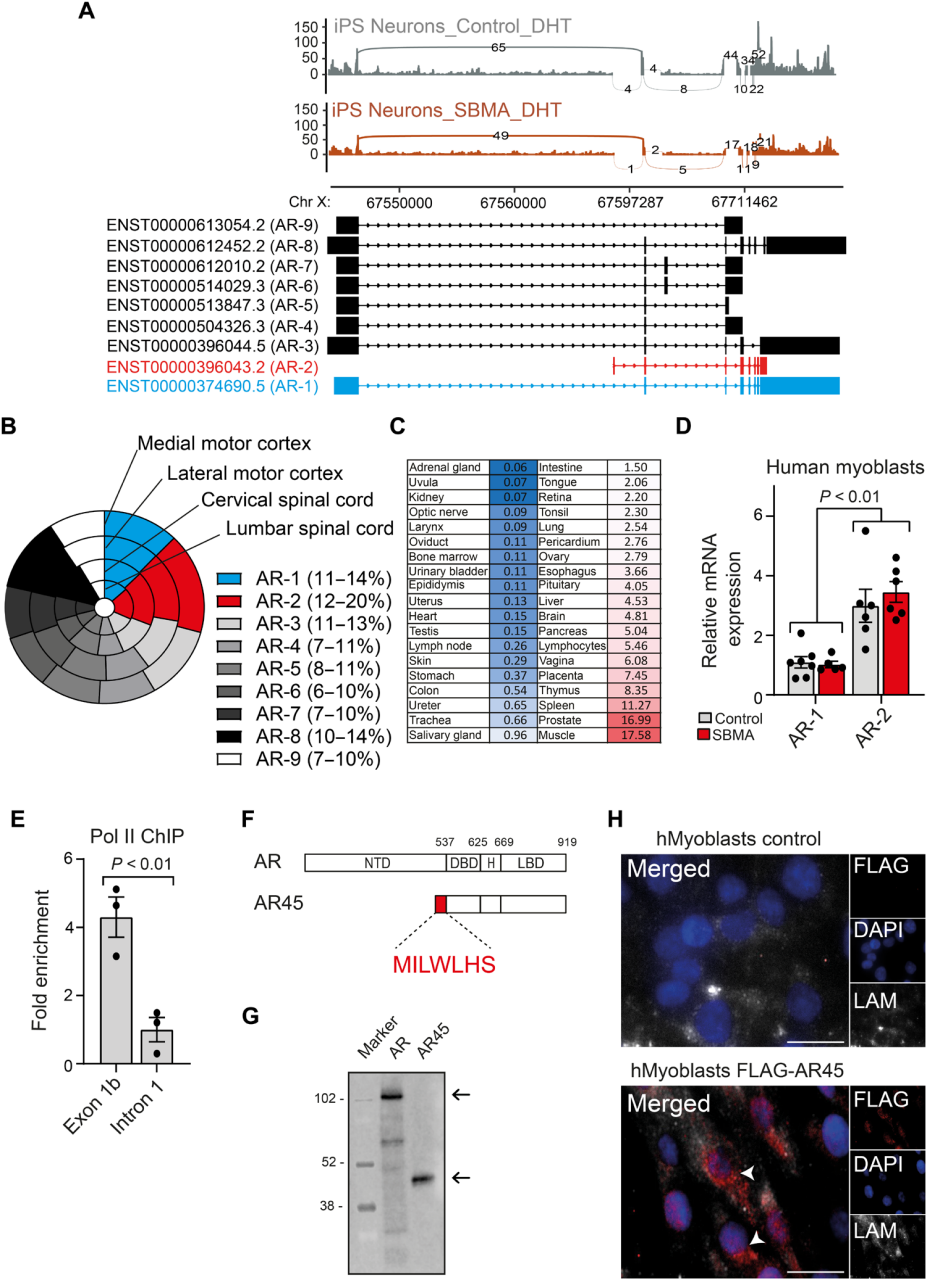
Gene expression profiling of the AR-2 isoform. (**A**) Sashimi plot depicting alternative isoforms for the *AR* gene generated from RNA-seq data in iPS-derived motor neurons from unaffected (control) and SBMA individuals. The number in the line represents the reads crossing a particular splice junction (curved lines). A scheme showing AR-1 (blue), AR-2 (red), and the other annotated AR isoforms is shown below. (**B**) Nested pie chart shows the proportion of mRNA *AR* isoforms levels from postmortem human tissues expressed as log-normalized counts. Percentage range for each isoform is in the parenthesis. (**C**) Heatmap indicating relative AR-2 transcript levels in human tissues normalized to *GAPDH*. (**D**) Relative mRNA expression levels normalized to *GAPDH* of AR-2 compared to AR-1 in human myoblasts from SBMA and unaffected subjects. (**E**) Chromatin immunoprecipitation (ChIP) from AR45-transfected MCF7 cells using anti–Pol II antibody shows increased occupancy on exon 1b of *AR* gene compared to a downstream sequence. (**F**) AR and AR45 protein domain conservation is shown. H, Hinge domain. Amino acid numbers refer to full-length (FL) human AR. Sequence of AR45 NTD is shown in the magnification. (**G**) Immunoblotting using an anti–C-terminal AR antibody of human embryonic kidney (HEK) 293 whole cell lysates where AR or AR45 was overexpressed. Size corresponding to the two isoforms (arrows) is expressed in kilodalton and displayed next to the protein marker. (**H**) Immunofluorescence micrographs of DHT-treated unmodified (control) or expressing endogenous FLAG-tagged AR45 (FLAG-AR45) human myoblasts. Cells were stained with anti-FLAG antibody (red), anti-laminin antibody (LAM; white), and 4′,6-diamidino-2-phenylindole (DAPI) (blue). Arrowheads indicate the AR45 signal. Scale bars, 20 μm. Data in (D) and (E) are means ± SEM. Each dot represents one replicate.

We first sought to determine the physiological expression levels of AR isoforms in human tissues that are relevant to SBMA pathogenesis. From our deep RNA sequencing (RNA-seq) data (~100 million per reads), obtained from DHT-treated induced pluripotent stem (iPS) cell–derived motor neurons (*n* = 3 control lines, *n* = 3 SBMA lines) and postmortem human medial and lateral motor cortexes, as well as cervical and lumbar spinal cords (*n* = 6 controls, *n* = 1 SBMA for each), we identified nine alternative AR transcripts expressed at low levels and encoding isoforms with various exon combinations (Fig. 1, A and B). Of these, AR-2 (ENST00000396043.2; CCDS43965.1) uniquely arises from the use of an alternative transcription start site (TSS) downstream of AR-1 TSS, and inclusion of exon 1b, located 22.1-kb downstream of exon 1 (Fig. 1A) (*25*). Similar distribution of alternatively spliced AR isoforms was observed for RNA-seq from iPS-derived motor neurons of SBMA individuals with or without DHT (Fig. 1A and fig. S1A). AR-2 accounts for an average of 17.4% of the AR isoforms in human brain (Fig. 1B). In a comprehensive human cDNA array, the highest relative AR-2 expression was found in prostate and skeletal muscle, which are androgen-responsive tissues (Fig. 1C). AR-2 transcript levels were higher than canonical AR-1 in human myoblasts from patients with SBMA and sex- and age-matched controls with no difference between the two groups (Fig. 1D). This chromatin locus is functionally accessible and actively transcribed, as indicated by increased deoxyribonuclease I hypersensitivity in 125 different cell types and transcription factor occupancy around exon 1b [from Encyclopedia of DNA Elements (ENCODE) projects] (fig. S2A) and RNA polymerase II enrichment relative to a downstream intronic sequence, assayed by chromatin immunoprecipitation (ChIP) (Fig. 1E). Multiple sequence analysis of exon 1b and the flanking regions suggest a close to 100% homology with *Rhesus macaque* and poor conservation in *Mus musculus* and *Rattus norvegicus*, where a stop codon downstream of the start codon infers that AR-2 isoform is not present in these species (fig. S2A) (*26*). The encoded protein contains a unique seven–amino acid sequence in the N terminus in place of the canonical NTD (Fig. 1F) and has a molecular mass of 45 kDa, hence the designated name AR45 (Fig. 1G). AR45 is expressed both in the cytoplasm and nucleus of human myoblasts, engineered to express the endogenously tagged AR45 protein, where an in-frame FLAG tag was inserted 5′ of the AR-2 gene by CRISPR-Cas9 method (Fig. 1H).

### AR45 regulates AR transcriptional activity

**Fig. 2:**
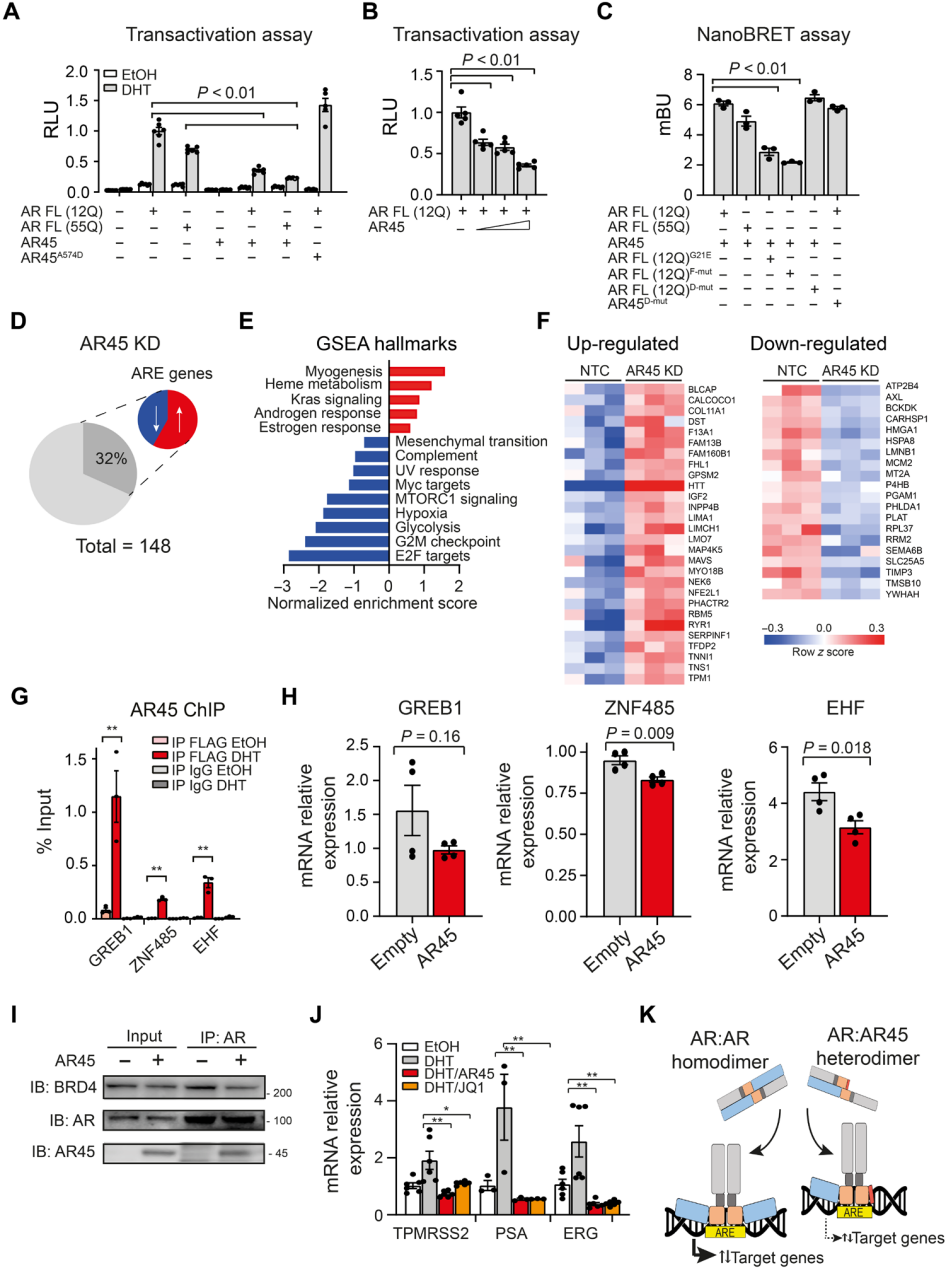
AR45 regulates AR transcriptional activity. (**A**) AR transactivation, expressed as relative luciferase unit (RLU), was measured in HEK293T cells transfected with vectors expressing FL AR or AR45, and pARE-E1b-Luc and β-galactosidase pCMVβ reporter constructs. (**B**) AR transactivation was measured in HEK293T cells expressing AR FL alone or with increasing concentration of AR45 (0.25, 0.5, and 1 μg). (**C**) AR45 and AR interaction was measured in HEK293T cells by bioluminescence resonance energy transfer (BRET) and expressed as milliBRET units (mBU). (**D**) Pie chart shows the proportion of up-regulated (58%; red) and down-regulated (42%; blue) genes containing ARE in their promoters among the differentially expressed transcripts upon AR45 knockdown (AR45 KD) in human myoblasts. (**E**) Gene set enrichment analysis (GSEA) using the Molecular Signature Database showing up-regulated (red) and down-regulated (blue) pathways upon AR45 KD. UV, ultraviolet; MTORC1, mechanistic target of rapamycin complex 1; G2M, G2 to M phase. (**F**) Heatmap of variance-stabilized RNA-seq read counts (*z* score) of ARE-containing transcripts. NTC, nontargeting control. (**G**) ChIP from FLAG-AR45–expressing MCF7 cells with anti-FLAG antibody or immunoglobulin G (IgG) isotype control. EtOH, ethanol. (**H**) mRNA expression levels normalized to *GAPDH* of known ARE-containing genes in DHT-treated AR45-overexpressing MCF7 cells. GREB1, growth regulating estrogen receptor binding 1; ZNF485, zinc finger protein 485; EHF, ETS homologous factor. (**I**) MCF7 cells nuclear extracts were subjected to IP using an anti–N-terminal AR antibody and immunoblotted for BRD4, AR, and AR45. One percent total lysate was used as input. Size is expressed in kilodalton and displayed next to the cropped blots. IB, immunoblot. (**J**) mRNA expression levels of known AR/BRD4 coregulated genes normalized to *GAPDH* in MCF7 cells upon treatment with DHT and bromodomain inhibitor JQ1 (500 nM) or AR45. ERG, ETS-related gene. (**K**) Working model: In addition to forming AR:AR homodimers, AR also heterodimerizes with its shorter isoform AR45, resulting in modulation of key AR transcriptional functions. Data in (A) to (C), (G), (H), and (J) are means ± SEM. Each dot represents one replicate. **P ≤ 0.01.

We next went on to characterize the role of AR45 on both wild-type and mutant AR biology. AR45 alone was not able to activate transcription of a luciferase reporter containing the AR-regulating prostate-specific antigen promoter (Fig. 2A). AR45 overexpression led to suppression of transactivation of both wild-type and mutant full-length AR in a dose-dependent manner (Fig. 2, A and B), an effect that was abolished by introducing a DBD-inactivating substitution A574D into the AR45 transgene (Fig. 2A), suggesting that the effect of AR45 on AR transactivation is specific and requires binding to DNA. AR dimerization through N/C-terminal interactions via the FxxLF motif and DBD/DBD interactions via the dimerization box (D-box) is a necessary step to activate the transcription of AR target genes (*27*). Since the DBD and LBD are conserved in AR45, we hypothesized that AR can also form heterodimers with AR45. We tested this hypothesis by using a bioluminescence resonance energy transfer (BRET) assay, which allows real-time detection of protein-protein interactions by exploiting the naturally occurring phenomenon of energy transfer from a donor enzyme to an acceptor fluorophore following enzyme-mediated oxidation of a substrate (*28*). All combinations of N- and C-terminal fusion constructs (fig. S3A) were transfected into AR-null cells, and one of the combinations exhibiting the highest BRET signal was chosen for further analysis (fig. S3B). BRET saturation curve indicated specific interaction between AR45 and full-length AR (fig. S3C). AR45 also interacts with full-length mutant AR (AR55Q). Mutation in the FxxLF motif (F-mut: F23, 27A/L26A) or N terminus of this motif (G21E) and not the D-box motif (D-mut: A596T/S597T) inhibited the AR/AR45 dimerization (Fig. 2C). We also found that AR45 forms homodimers (figs. S3D and S4E). The AR/AR45 interaction was further validated by co-immunoprecipitation (co-IP) (fig. S4A). AR protein levels were not affected by the presence of AR45 in a cycloheximide chase experiment, suggesting that AR45 does not alter AR protein turnover (fig. S4, B and C).

To gain insight into endogenous AR45 functions, we next profiled global gene expression changes via RNA-seq in DHT-treated human myoblasts where AR-2 transcript was knocked down (KD) compared to control cells treated with a nontargeting siRNA. We first designed an array of 11 small interfering RNAs (siRNA), spanning exon 1b sequence (fig. S5A). To improve their pharmacokinetic properties, these siRNAs were fully chemically modified and lipid-conjugated (*29*). These siRNAs resulted in selective silencing of endogenous AR-2 (ranging from 45 to 90%) (fig. S5B). AR-2 KD in human myoblasts using the best-performing siRNA was confirmed by reverse transcriptase quantitative polymerase chain reaction (RT-qPCR) before RNA-seq (fig. S6A). Using differential expression analysis (DEseq; *P* < 0.05, fold change >1.5), we identified 148 genes that were differentially expressed upon AR-2 KD with higher proportion of up-regulated genes (59%) (table S1). Of the differentially expressed genes, 32% were found to contain an ARE sequence in the promoter region (Fig. 2D and table S1). Gene set enrichment analysis (GSEA) showed up-regulation of genes associated with AR and estrogen response (Fig. 2E). Among the ARE-containing genes, endogenous AR-2 KD resulted in up-regulation of *NFE2L1* (Nuclear factor, erythroid 2 like 1), a master regulator of proteasome subunits, and down-regulation of *HSPA8* (Heat shock protein family A member 8), a member of the chaperone-assisted selective autophagy complex, suggesting that AR45 may be involved in the regulation of androgen-induced cell stress signals (Fig. 2F) (*30*, *31*).

To investigate whether AR and AR45 co-occupy the same genomic loci, we carried out ChIP analyses at the promoter regions of *GREB1*, *ZNF485*, and *EHF*, which are known as AR direct target genes (fig. S7A) (*32*). We detected signal enrichment of FLAG-tagged AR45 (FL-AR45) upon DHT treatment (Fig. 2G), indicating AR45 occupancy at the AR binding sites. Overexpression of AR45 resulted in reduced transactivation of those targets (Fig. 2H).

To elucidate the underlying mechanism of the effect of AR45 on AR transcriptional activity, we investigated whether overexpression of AR45 affects the interaction between AR and its transcriptional coregulators. In a co-IP assay in human breast cancer MCF7 (Michigan Cancer Foundation-7) cells, we demonstrated that AR45 reduces binding affinity between AR and the master transcriptional regulator BRD4 (Bromodomain-containing protein 4) (Fig. 2I and fig. S8A). Treatment of these cells with 500 nM JQ1, a selective small-molecule BRD4 inhibitor, recapitulated the decreased expression of AR target genes observed with AR45 overexpression, further validating the functional relevance of this finding (Fig. 2J). Together, these results demonstrate that AR45 forms heterodimers with both wild-type and mutant AR and modulates AR genomic activity by reducing binding to its transcriptional coregulators (Fig. 2K).

### Viral delivery of AR45 ameliorates the disease phenotype in a mouse model of SBMA

On the basis of previous studies showing that AR binding to its target genes through the DBD is critical to SBMA pathogenesis (*17*) and that disrupting the interaction between AR and its transcriptional coregulators represents a promising therapeutic strategy for this disease (*17*, *22*, *23*, *33*), we reasoned that overexpression of AR45 would be able to counteract SBMA toxicity.

**Fig. 3:**
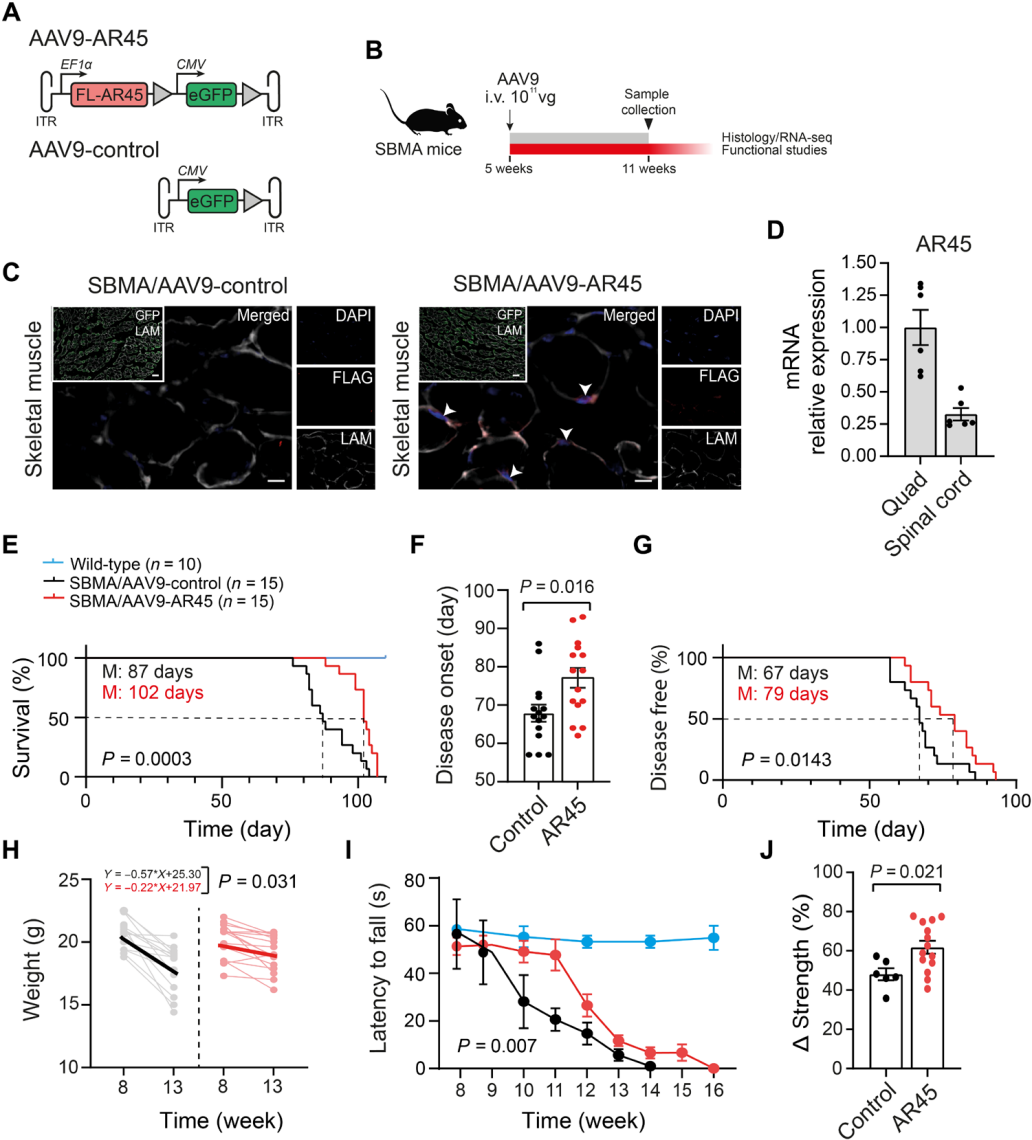
AAV9-mediated delivery of AR45 improves the SBMA phenotype. (**A**) Schematic representation of AAV vectors used in this study. AAV inverted terminal repeats (ITR) and SV40 poly-adenylation signal (triangles) are also indicated. (**B**) Experimental design; arrow indicates the timing of the intravenous (i.v) injection. Dose is expressed as vector genomes (vg). (**C**) Representative images of skeletal muscle from SBMA mice treated with AAV9-AR45 or AAV9-control, stained with FLAG antibody (red), LAM (white), GFP (green), and DAPI (blue). Arrowheads indicate AR45 signal. Scale bars, 100 μm (merged) and 10 μm (inset). (**D**) AR45 mRNA expression levels normalized to *GAPDH* in spinal cord relative to quadriceps muscle of treated SBMA mice. (**E**) Kaplan-Meier survival estimation of SBMA mice treated with AAV9-control or AAV9-AR45 (M, median; log-rank test). Survival of wild-type mice is shown for reference. (**F** and **G**) AR45-treated SBMA mice show a delay in the disease onset by 12 days in average (M, median; log-rank test) compared to their SBMA littermates. (**H**) Weight loss is significantly reduced in AR45-treated SBMA mice. Weights for each mouse at the beginning and end of the study are shown in gray (AAV9-control) and pink (AAV9-AR45). (**I**) Rotarod performances, expressed as latency to fall, are compared between treatment groups [two-way analysis of variance (ANOVA)]. Performances of wild-type mice are also shown for reference. (**J**) Average percentage reduction in grip strength at the end of the study (week 13) compared to disease onset (week 8) in SBMA mice treated with AAV9-control and AAV9-AR45 is displayed. Data in (D), (F), (I), and (J) are means ± SEM. Each dot represents one replicate.

We generated two adeno-associated viruses (AAVs) carrying single-stranded AAV9 vectors encoding either FL-AR45 cDNA driven by the ubiquitous elongation factor 1α (EF1α) promoter and enhanced green fluorescent protein (eGFP) driven by cytomegalovirus (CMV) promoter (AAV9-AR45) or eGFP only (AAV9-control) (Fig. 3A). The SBMA model chosen for the study expresses the human AR transgene with 100 glutamine residues (AR100Q) and recapitulates the main features of the SBMA phenotype, with androgen-dependent muscle atrophy, reduced grip strength, denervation, and shortened survival, starting at 7 weeks of age (*34*). Notably, protein levels of monomeric human AR in the skeletal muscle and spinal cord of these mice are comparable to the wild type’s (*34*). On the basis of quantitative measures of body weight, we performed a statistical power analysis to establish the minimum number of mice required for each cohort in the trial (Cohen’s *d* effect size: 0.8; minimum sample size for each group: 10 mice). Male transgenic SBMA mice were blindly randomized to receive one of the two AAVs by single tail-vein injection at 5 weeks of age, at a dose of 2 × 10^11^ to 2.5 × 10^11^ vector genomes (vg), in two experiments, aimed at assessing phenotypic/functional (*n* = 15) and histopathological/molecular (*n* = 3) differences between the two groups (Fig. 3B). For translational relevance, the timing of injection was chosen so that the peak of transgene expression matched the appearance of the disease manifestations in this model (*35*). In quadriceps muscle tissues collected 6 weeks after injection, we observed ~60% GFP-positive fibers and AR45 staining in the treatment group, indicating efficient viral transduction (Fig. 3C). Relative expression of viral-delivered *AR45* mRNA in lumbar spinal cord was ~4 times lower than quadriceps muscle (Fig. 3D), consistent with reduced central nervous system transduction after peripheral injection of single-stranded AAV9 vectors in adult mice (fig. S9) (*36*). AR45 treatment significantly prolonged the life span of these mice from a median of 87 to 102 days (primary outcome measure; Fig. 3E) and delayed the disease onset by 12 days, determined as the age at which the mice start losing body weight (Fig. 3, F and G). Notably, AR45 overexpression reduced end-of-study body weight loss (Fig. 3H and fig. S10A) and improved rotarod activity (Fig. 3I) and rear grip strength (Fig. 3J and fig. S10B). Together, these findings demonstrate that viral delivery of AR45 ameliorates the disease phenotype in SBMA mice.

### AR45 treatment restores the pathologic degeneration and transcriptional profile in SBMA mice without decreasing inclusion accumulation

**Fig. 4:**
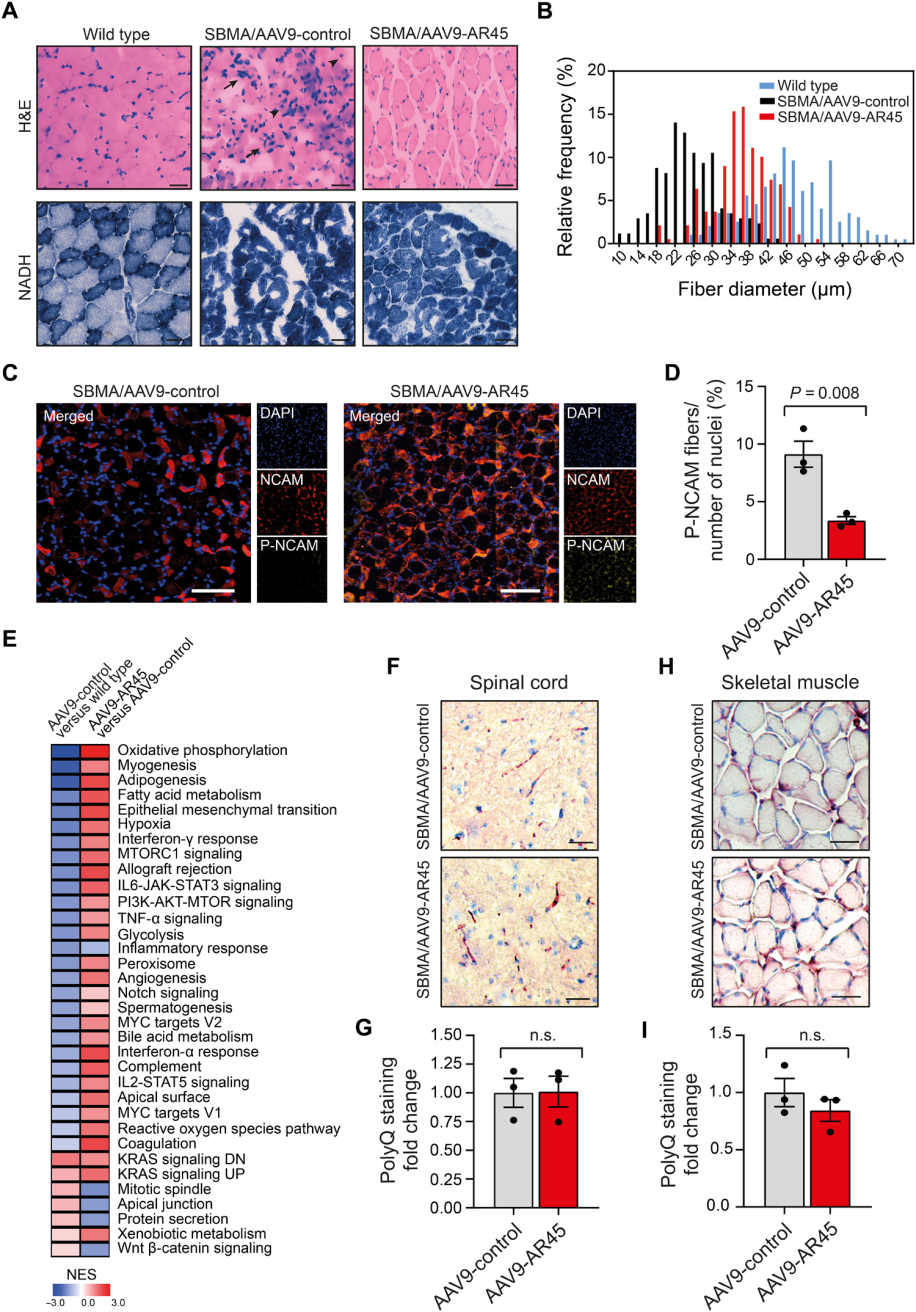
AR45 treatment restores the SBMA-associated degeneration in mice. (**A**) Representative cross sections of gastrocnemius muscle from 11-week-old wild-type and SBMA mice treated with AAV9-control or AAV9-AR45. Sections were stained with H&E and NAD/NADH for muscle morphology. Arrows indicate atrophied myofibers. Arrowheads indicate myofibers containing centralized nuclei. Scale bars, 100 μm. (**B**) Relative frequency distribution of myofiber cross-sectional diameter size sorted in wild-type, control treated-, and AR45-treated SBMA mice. (**C**) Representative cross sections of gastrocnemius muscle from 11-week-old SBMA mice treated with AAV9-control or AAV9-AR45 stained with neural cell adhesion molecule (NCAM) antibody, polysialic acid–NCAM (P-NCAM) antibody, and DAPI. Scale bars, 100 μm. (**D**) Quantification of P-NCAM/NCAM colocalized regions relative to the total number of nuclei is graphed. Quantification was performed from a total of six fields for all groups, three mice per treatment group. (**E**) Heatmap shows the normalized enrichment scores (NES) of gene sets significantly enriched at *P* < 0.05 and false discovery rate ≤ 0.25 in the tibilias anterior muscle of AR45-treated compared to control SBMA mice with overexpressed gene sets in red and underexpressed gene sets in blue. Thirty-two pathways were partially restored upon AR45 overexpression. (**F**) Representative cross sections of lumbar spinal cord and (**H**) gastrocnemius muscle from 11-week-old wild-type and SBMA mice treated with AAV9-control or AAV9-AR45, stained with 1C2 antibody and hematoxylin. Scale bars, 100 μm. (**G** and **I**) Quantification of the number of 1C2-positive staining normalized to the number of cells. Data are means ± SEM. Quantification was performed from a total of six fields for all groups, three mice per treatment group. n.s., not significant.

To further characterize the effects of AR45 treatment on SBMA, quadriceps muscles from 11-week-old SBMA mice treated with AAV9-AR45 or AAV9-control, and wild-type littermates were collected for histopathological analyses. Staining of quadriceps muscle cross sections with hematoxylin and eosin (H&E) and nicotinamide adenine dinucleotide (NADH) showed a marked amelioration of the muscle architecture, with reduced angulated myofibers, grouped atrophic fibers, enlarged fibers with central nuclei (Fig. 4A), and increased mean cross-sectional myofiber areas (Fig. 4B). Furthermore, we observed marked reduced colocalization of neural cell adhesion molecule (NCAM)/polysialic acid–NCAM (P-NCAM) staining, indicating decreased denervation/re-innervation activity, in AR45-treated SBMA mice compared to transgenic controls (Fig. 4, C and D). To explore whether AR45 treatment restores the disease-related transcriptional dysregulation, we performed RNA-seq in tibialis anterior (TA) muscle from 11-week-old transgenic male mice treated with AAV9-AR45 or AAV9-control and wild-type littermates.

We identified 6644 significantly dysregulated genes in SBMA skeletal muscle compared to wild type (3354 up-regulated and 3290 down-regulated) (fig. S11A and table S2), with changes in genes involved in several functions, including mitochondrial homeostasis, lipid metabolism, glycolysis, p53 signaling, and DNA repair, as revealed by the functional network and pathway annotation (fig. S11B). These alterations overlap with published transcriptomic profiles using other SBMA models (*37*–*40*). Notably, AR45 treatment resulted in significant restoration of 27 of the 32 down-regulated (84%) and 5 of the 17 up-regulated (29%) SBMA hallmark molecular signatures (Fig. 4E), with gene expression clusters showing a pattern toward normalization (fig. S11C and table S3). Treatment did not affect serum testosterone concentrations in mice (fig. S12A). In addition, no change in human or mouse AR mRNA and protein levels (fig. S12, B to D), as well as in the polyQ-positive inclusions and ubiquitin staining in both spinal cord (Fig. 4, F and G, and fig. S13A) and skeletal muscle (Fig. 4, H and I, and fig. S13B), was observed upon AR45 overexpression, supporting a model where therapeutic benefit can be achieved independently of polyQ AR aggregation.

### Systemic delivery of AAV9-AR45 vector did not induce detectable toxicity in SBMA mice

**Fig. 5:**
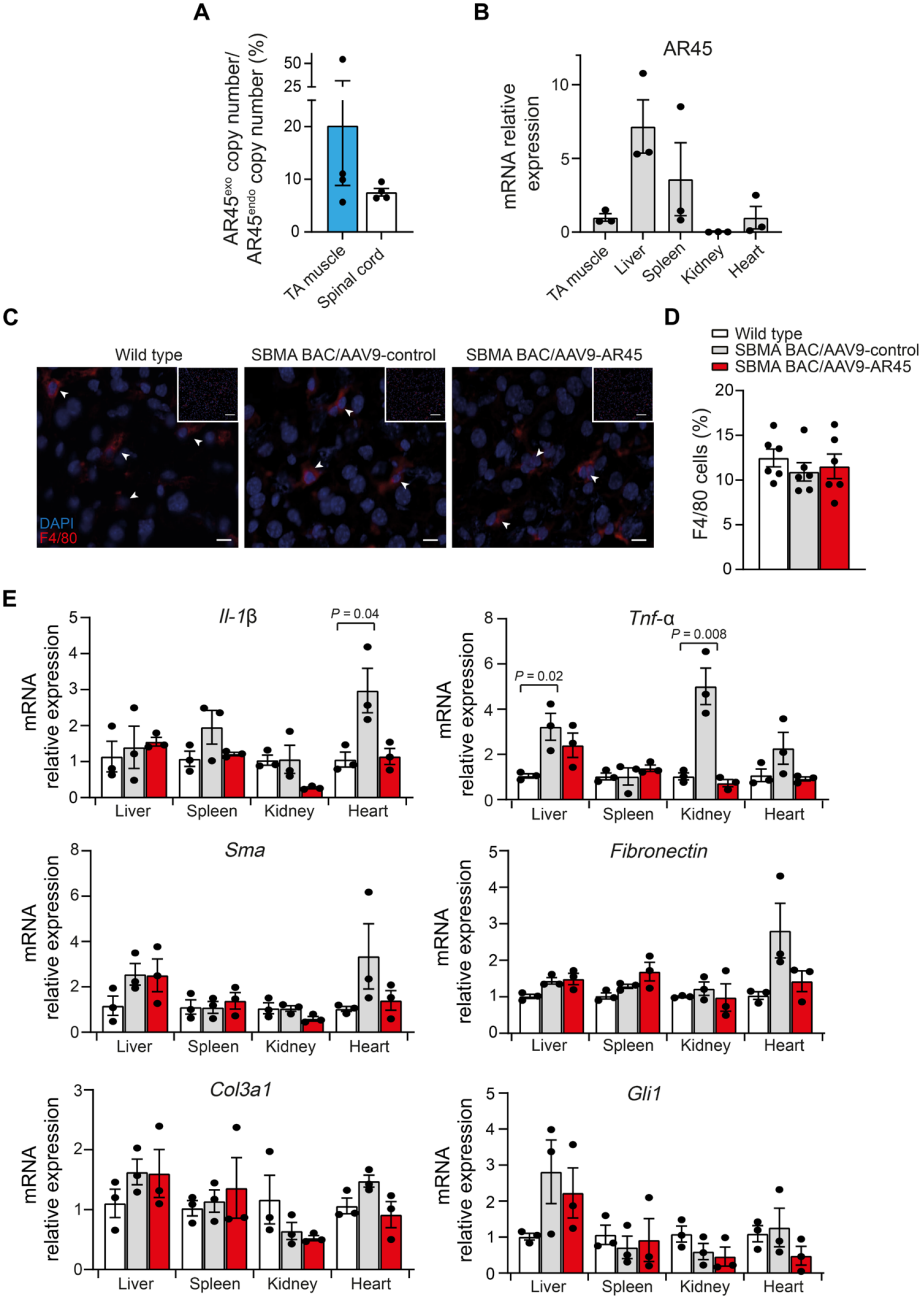
Systemic delivery of AAV9-AR45 did not induce detectable toxicity in mice expressing AR-2 at endogenous levels. (**A**) Ratios of exogenous (exo) to endogenous (endo) AR45 mRNA copy number per ng of total RNA in TA muscle and lumbar spinal cord of four SBMA BAC transgenic mice 5 weeks after treatment with AAV9-AR45 (10^11^ vg) are shown. (**B**) mRNA expression levels of exogenous AR45 in tissues of mice treated with AAV9-AR45 normalized to *GAPDH* housekeeping gene and expressed as fold change to levels in TA muscle are shown. Data are means ± SEM. Each dot represents one replicate (*n* = 3). (**C**) Representative cross sections of liver from adult wild-type and SBMA BAC transgenic mice treated with AAV9-control or AAV9-AR45 stained with F4/80 antibody and DAPI. Arrowheads indicate the F4/80 positive cells. Scale bars, 100 μm (low magnification) and 10 μm (high magnification). (**D**) Number of F4/80-positive cells per 100 cells is graphed. Quantification was performed from a total of six fields for all groups, three mice per treatment group. (**E**) mRNA expression levels of inflammatory and fibrotic marker genes in tissues from wild-type and SBMA BAC transgenic mice treated with AAV9-control or AAV9-AR45 normalized to *GAPDH* housekeeping gene and expressed as fold change to levels in the respective wild-type tissues are shown. Data are means ± SEM. Each dot represents one replicate (*n* = 3).

To investigate the potential undesirable effects of both viral vector and AR45 transgene overexpression on vital physiological functions, we used the bacterial artificial chromosome (BAC) SBMA transgenic mouse model, which develops a very mild neuromuscular phenotype, unsuitable for proof of concept studies but, by harboring the entire length of the human *AR* locus (*41*, *42*), uniquely expresses AR45 at physiological levels. Using the same treatment paradigm adopted for the efficacy study, 5-week-old male mice were injected by tail vein with AAV9-AR45 or AAV9-control, at a dose of 2 × 10^11^ to 2.5 × 10^11^ vg and sacrificed after 6 weeks before the disease onset of this model (*41*). Copy number analysis per nanogram of total RNA in four mice revealed an average of 20.2% increase of AR45 expression over endogenous levels in TA muscle and 7.5% in lumbar spinal cord upon treatment with AAV9-AR45 vector (Fig. 5A) and highest vg concentration in the liver and spleen relative to the skeletal muscle (Fig. 5B), consistent with previous studies evaluating AAV9 transduction properties in vivo (*43*).

Histopathologic analysis of the heart, spleen, liver, skeletal muscle, kidney, brain, and testis revealed no adverse macroscopic findings. We did not observe increased infiltration of F4/80-expressing macrophages (Fig. 5, C and D) in the liver of SBMA mice treated with AAV9-control or AAV9-AR45 compared to wild-type littermates. A mild elevation in *Il-1*β and *Tnf-*α mRNA levels was observed in tissues of SBMA mice (Fig. 5E), further confirming a role of AR in transcriptionally inducing an inflammatory response (*44*). Notably, AR45 treatment normalized this effect (Fig. 5E). To assess whether the treatment results in chronic inflammation and fibrosis, we analyzed transcripts levels of critical markers (*Sma*, *Fibronectin*, *Col3a1*, and *Gli1*), which were unchanged in treated SBMA mice compared to untreated wild-type littermates (Fig. 5E). Collectively, these data suggest the lack of deleterious effects against the AAV9 or the AR45 transgene.

## DISCUSSION

AR-mediated transcription is dependent on coregulatory proteins to recruit transcriptional machinery, increase chromatin accessibility, and enhance transactivation (*45*). PolyQ AR–altered genomic activity has been shown to be essential to trigger disease pathogenesis in SBMA (*17*). Therapeutic strategies selectively targeting mutant AR transcriptional dysregulation by modulating the actions of coregulators, without affecting other critical nongenomic functions of AR, have a competitive advantage over existing approaches, which are yet to be translated into a treatment for patients with SBMA. Multiple nuclear receptor isoforms arise from a single gene by alternative splicing or by the use of alternative promoters in health and disease to regulate their genomic and nongenomic activities, and AR is no exception (*46*). Pathological AR protein variants, yielding from gene alterations and abnormal splicing patterns, have been identified in human prostate cancer (*47*, *48*) and have been shown to confer resistance to androgen therapies (*48*, *49*), providing opportunities for the identification of promising therapeutic targets and prognostic markers (*50*–*52*). Naturally occurring AR isoforms in healthy tissues are less well characterized. Here, we have identified nine alternative transcript AR variants in iPS-derived motor neurons and human brains. Of these, AR-2 appears to be the most abundantly expressed, resulting from inclusion of the alternative exon 1b, located in intron 1 of the *AR* gene, and encoding a truncated AR variant with a unique seven–amino acid peptide in place of the canonical NTD harboring the polyQ stretch, 45 kDa in size, hence the name AR45 (*25*). AR45 was previously found to be widely expressed in human tissues to interact with AR in two-hybrid assays and inhibit proliferation when overexpressed in prostate cancer LNCaP (Lymph Node Carcinoma of the prostate) cells (*25*). Our study—by using deep RNA-seq to provide quantitative expression profiles in human tissues, assessing the transcriptome-wide effects of AR-2 silencing, and measuring the protein-DNA interaction by ChIP assay at ARE sites—significantly expands on these findings, supporting a model where AR45 acts as an endogenous modulator of AR transcriptional activity. Overexpression of AR45 in cells resulted in reduced transactivation of ARE-containing AR target genes in a dose-dependent manner by reducing AR binding to its transcriptional coactivator BRD4. BRD4, a member of the bromodomain and extraterminal (BET) protein family, is a master transcriptional and epigenetic regulator that plays a pivotal role during several processes from embryogenesis to cancer development. Upon interaction with chromatin-associated transcription factors, BRD4 is recruited to hyperacetylated histone regions along the chromatin and promotes transcription initiation and elongation (*53*–*55*). By physically interacting with AR NTD, BRD4 plays a critical role in facilitating AR-mediated transcription (*56*, *57*); clinical evaluation of BET inhibitors is currently underway in castration-resistant prostate cancer (clinical trial: NCT03035591).

These findings prompted us to test delivery of this isoform to treat SBMA via AAV9. This viral vector is the serotype of choice to transduce neurons and skeletal muscle (*58*).

To simulate a trial in early disease–stage patients, mice were treated at 5 weeks of age with a single-stranded genome AAV9 vector (2 × 10^11^ vg): Because of the rate-limiting step involving the de novo synthesis of the second DNA strand, this treatment regimen results in a 2-week-delayed transgene expression, corresponding to the appearance of disease manifestations in this model, as previously documented (*34*, *35*). Consistent with previous biodistribution studies after peripheral intravascular delivery of AAV9 vectors in adult mice (*36*), we observed extensive transduction in skeletal muscle, with fourfold-reduced transgene levels in lumbar spinal cord 6 weeks after treatment. Scaling this dose up to improve neuronal transduction for human testing would represent a manufacturing challenge and may result in a significantly increased risks of liver toxicity in patients, as emerged in clinical trials using high dose of AAV8 (3 × 10^14^ vg/kg) to deliver Audentes Therapeutics’ AT132 for patients with X-linked myotubular myopathy (*59*) (clinical trial: NCT03199469). Notably, in the last two decades, an increasing number of observations derived both from preclinical and clinical studies have indicated that skeletal muscle is a primary driver of SBMA pathogenesis (*16*, *60*–*66*). Furthermore, skeletal muscle–specific excision of polyQ AR in a BAC mouse model featuring a floxed AR first exon is sufficient to prevent the development of disease manifestations (*41*), and peripherally administered treatments that do not cross the blood-brain barrier are able to ameliorate the neuromuscular phenotype in mice (*21*, *64*, *67*), overall suggesting that muscle is a target for therapy development in SBMA (*68*).

More than 120 gene therapy clinical trials using AAV-based vectors are currently in use in numerous gene therapy clinical trials, ranging from hemophilia to inherited blindness, with reports of benefit for patients and favorable safety profiles with a transient and asymptomatic hepatitis as the most severe side effect. In 2019, the U.S. Food and Drug Administration approved AveXis’ ZOLGENSMA (Onasemnogene abeparvovec) for the treatment of patients less than 2 years of age with spinal muscular atrophy using an AAV9 vector at 1.1 × 10^14^ vg/kg by intravascular administration (clinical trials: NCT02122952, NCT 03306277, NCT03421977, and NCT03505099), paving the way for the development of AAV-based therapeutics for other neuromuscular conditions.

By characterizing the role of a naturally occurring AR isoform in AR homeostasis, here we provide proof-of-principle evidence of efficacy and safety of a gene therapy strategy based on AAV9-mediated delivery of the AR variant AR45 in SBMA mice, with high potential to be translated into a treatment for patients. The slightly less notable therapeutic effect observed, compared with previously published repeated systemic antisense oligonucleotide administrations (*21*), could be partly attributed to the more severe SBMA models used in this work and to the low AAV9 dose used for single peripheral injection. Before bringing this strategy into clinical testing, many aspects of AAV biological properties, which cannot be accurately predicted in preclinical studies, such as vector immunogenicity, therapeutic potency, duration of expression, and potential toxicity, will have to be further elucidated (*69*), particularly in the context of adult-onset neuromuscular diseases, progressing over the course of years, like SBMA. Given the critical importance of AR transcriptional control in various diseases such as androgen insensitivity syndrome, benign prostatic hyperplasia, and prostate cancer (*70*), intriguingly, this isoform may potentially be a target for therapy not only for SBMA but also for other AR-related conditions.

## MATERIALS AND METHODS

### Mice

Experiments were carried out in the Biomedical Sciences Unit, University of Oxford according to procedures authorized by the U.K. Home Office (Animal Scientific Procedures Act 1986). Mice were housed in individually ventilated caging systems, with access to food and water ad libitum. Only male mice were used in the analyses. AR100Q (*71*) and BAC AR121Q (*41*) transgenic animals were provided by the Pennuto laboratory and the La Spada laboratory, respectively. Dual promoter AAV vector plasmids containing an expression cassette consisting of a human EF1α promoter followed by AR45 cDNA or mock sequence and human CMV promoter followed by cDNA-encoding GFP were provided by SignaGen Laboratories (Rockville, MD). A viral load of 2 × 10^11^ vg was injected into the tail veins of SBMA AR100Q mice. Injection volume was brought to 100 μl with 1× phosphate-buffered saline (PBS). The body weight, rotarod, and grip strength using hanging wire and strength meter (Bioseb) were recorded weekly. Randomization was performed among littermates, the treatments were administered, and analyses were performed by blinded investigators. Study end-point is set at a body weight loss of >20%.

### Cell lines and reagents

Human embryonic kidney (HEK) 293T and MCF7 cells were obtained from the American Type Culture Collection (ATCC) and cultured as described on the ATCC cell culture guide. Immortalized human myoblast cells (MRC CNMD Biobank London, L954/1284 M-I) were maintained in skeletal muscle cell growth medium (PromoCell) supplemented with supplement mix (PromoCell), 10% (v/v) fetal bovine serum (FBS; Gibco), 1× GlutaMAX (Gibco), and 1× antibiotic antimycotic (Gibco). iPS cells were cultured and differentiated into motor neuron–like cells in the Fischbeck laboratory, as described previously (*72*). All cell lines were cultured at 37°C and 5% CO_2_. AR induction was performed by addition of DHT (Sigma-Aldrich) to a final concentration of 10 nM in the respective cell culture medium. An equal volume of ethanol was added for the uninduced controls.

### Human samples

Anonymized brain tissues (CGND_HRA_00063, CGND_HRA_00218, CGND_HRA_00224, CGND_HRA_00035, CGND_HRA_00236, CGND_HRA_00399, CGND_HRA_00400, CGND_HRA_00402, CGND_HRA_00411, CGND_HRA_00412, CGND_HRA_00654, CGND_HRA_00655, CGND_HRA_00657, and CGND_HRA_00091) were provided by the Target ALS Post-Mortem Tissue Core. These samples were acquired through various Institutional Review Board protocols from member sites and transferred to the New York Genomic Consortium in accordance with all applicable foreign, domestic, federal, state, and local laws and regulations for processing, sequencing, and analyses.

### Plasmids construction

To generate different constructs of AR FL (12Q), AR FL (55Q), and AR45, we PCR-amplified the AR FL (12Q), AR FL (55Q), and AR45 cDNAs from their respective expression construct and cloned the PCR amplicons separately into a mammalian expression vector under the control of the CMV promoter with or without an N-terminal FLAG epitope tag. To generate NanoBRET fusion constructs, these PCR amplicons were cloned separately into pFN21A HaloTag CMV Flexi, pFC14K HaloTag CMV Flexi, pFN31K Nluc CMV-neo Flexi, and pFC32K Nluc CMV-neo Flexi vectors via Sgf I and Pme I or Sgf I and Eco ICRI following the manufacturer’s protocol (Promega). The mutant constructs with mutations at the FxxLF motif (F23,27A/L26A) or (G21E), at the D-box (A596T/S597T), or at the DBD (A574D) of AR were generated by site-directed mutagenesis using the Q5 Site-Directed Mutagenesis Kit (New England BioLabs). All plasmids were sequence-verified.

### CRISPR-Cas9–based genome editing

To generate human myoblast cell lines expressing endogenously tagged AR45 for immunofluorescence studies, FLAG epitope tag was introduced to the N terminus of endogenous AR-2 gene in human myoblast cells via CRISPR-Cas9 genome editing strategy. A total of 5 × 10^5^ human myoblast cells were transfected with 1.2 μg of plasmid encoding both Cas9 protein and single-guided RNA targeting the start of AR-2 sequence and 0.8 μg of plasmid containing the 3× FLAG sequence flanked by 400–base pair (bp) arms of homology on either side of the insertion site. The latter served as DNA donor templates in the genome editing strategy, and it was synthesized by Invitrogen GeneArt Gene Synthesis service. Cas9-encoding pSpCas9(BB)-2A-GFP plasmid (px458) was a gift from F. Zhang (Addgene plasmid no. 48138). Transfection was performed by nucleofection using a Neon Transfection System (Thermo Fisher Scientific) following the manufacturer’s protocol. Cells were given three pulses of 1650 V for 10 ms. Cells were then cultured for 48 hours in complete medium lacking antibiotic antimycotic. Transfected cells that expressed Cas9-GFP were singularly sorted by fluorescence-activated cell sorting into 96-well culture dishes to establish clonal populations (Flow Cytometry Facility, Sir William Dunn School of Pathology, University of Oxford).

### Luciferase report assays

To determine the effect of AR45 on AR-dependent transcriptional activity, HEK293T cells were transiently transfected with Lipofectamine 2000 reagent (Invitrogen) in Opti-MEM I–reduced serum medium (Gibco) with 0.5 μg of pARE-E1b-luc (luciferase report vector with an insertion of an ARE upstream of the luc2 gene), 0.5 μg of pRL-TK (Renilla luciferase control) (Promega), and 0.5 μg of AR FL and/or AR45 vector plasmids. Following 24 hours of transfection, cells were washed and treated with DHT or an equal volume ethanol as negative control. Cells were harvested after 24 hours and firefly and Renilla luciferase substrates (Dual-Luciferase Reporter Assay, Promega) were added, and luciferase activity was measured using a microplate spectrophotometer (CLARIOstar, BMG LabTech) according to the manufacturer’s protocol. Renilla luciferase activity was used as the internal normalization control.

### NanoBRET assay

To investigate the interaction between AR FL protein and AR45 protein, NanoBRET assay was performed according to the manufacturer’s instructions (Promega NanoBRET Protein: Protein Interaction System). Briefly, HEK293T cells were cotransfected with a NanoLuc luciferase (Nluc) fusion plasmid and a HaloTag fusion plasmid with Lipofectamine 2000 reagent. A total of 200 ng of Nluc and 2000 ng of HaloTag fusion plasmid were used, unless otherwise stated. The combination of Nluc-MDM2 and p53-HaloTag was included as a positive control, and the combination of Nluc-MDM2 and HaloTag-SMAD4 was included as a negative control. At 24 hours after transfection, cells were detached with TrypLE Express Enzyme (Gibco) and resuspended in Opti-MEM I–reduced serum medium, no phenol red (Gibco), supplemented with 4% (v/v) FBS. Cells were counted and seeded in triplicate into a 96-well microplate at 2 × 10^5^ cells/ml. Cells were treated with or without 10 nM DHT. HaloTag NanoBRET 618 ligand or dimethyl sulfoxide (DMSO) (no-ligand control) were added to each well at 1:1000 dilution. At 24 hours, NanoBRET Nano-Glo substrate was added to the cells, and NanoBRET readings at 460 and 618 nm were obtained with a microplate spectrophotometer (CLARIOstar, BMG LabTech). Mean-corrected NanoBRET ratios in milliBRET unit were calculated using the following formula:

Mean-corrected NanoBret ratio, milliBRET =.

[(618 nmEM / 460 nmEM)Experimental − (618 nmEM / 460 nmEM)No-ligand control] × 1000.

### Chromatin immunoprecipitation

AR45 ChIP was conducted on MCF7 cells transiently transfected with FLAG-AR45 plasmids. Untransfected MCF7 cells were included as the negative controls. Cells were treated with 10 nM DHT 24 hours after transfection. At 48 hours, cells were cross-linked with 1% formaldehyde for 10 min and harvested and lysed. Cell lysates were sonicated to an average DNA fragment length of 200 to 400 bp. The experiments were conducted as described (*73*) using 300 μg of chromatin and 14 μg of anti-FLAG antibody (Sigma-Aldrich, F1804) or anti mouse immunoglobulin G (IgG) antibody (Cell Signaling Technology, 5415s). RNA Pol II ChIP was performed as described (*74*) on DHT-treated human myoblasts cells using Pol II antibody (2 μl per IP; Santa Cruz Biotechnology, sc-9001). Purified DNA samples were used for quantitative real-time PCR. Oligonucleotides used for the PCR are available in table S4.

### RNA and cDNA preparation and RT-qPCR

Ten milligrams of muscle, spinal cord, or liver tissues was homogenized in 1-thioglycerol/homogenization solution for 2 × 1 min using Precellys tissue homogenizer (Bertin Instruments). Total RNA was isolated from murine tissues or cell lines using Maxwell RSC simplyRNA tissue or cells kit and Maxwell RSC Instrument (Promega) according to the manufacturer’s protocol. One microgram of RNA was used for cDNA synthesis with the High-Capacity cDNA Reverse Transcription Kit (Applied Biosystems). RT-qPCR reactions were set up with fast SYBR green master mix or TaqMan gene expression master mix (Applied Biosystems) using 20 to 50 ng of DNA templates. The reactions were run with the default parameters of the Applied Biosystems Step One Plus real-time PCR system (for 96-well format). AR45 differential expression in normal human tissues was detected by RT-qPCR using the TissueScan cDNA Array (Origene, HMRT104). Oligonucleotides used in the RT-qPCR experiments are listed in table S2.

### AR45 siRNA screens and AR45 KD experiments

A panel of 11 siRNAs targeting all possible regions specific for AR45 transcript and one nontargeting control siRNA (NTT) were designed and synthesized as described (*75*, *76*). MCF7 cells were seeded and cultured at 10^5^ cells/ml on 12-well dishes overnight in Dulbecco’s modified Eagle medium (DMEM) with GlutaMAX supplement (Gibco) supplemented with 10% FBS and 1× antibiotic antimycotic solution. One micromolar of siRNA-AR45 or siRNA-NTT was added to MCF7 cells in 50:50 Opti-MEM I–reduced serum and DMEM cell culture medium in a final 3% (v/v) FBS. Total RNA was extracted from cells with TRIzol reagent according to the supplier’s protocol (Invitrogen). cDNA was prepared and RT-qPCR was performed as described in the previous section using FAM (Fluorescein amidites)-labeled TaqMan array Hs04272731_s1 specific for AR-1, Hs04275959_m1 specific for AR-2, and Hs99999905_m1 for GAPDH for normalization.

For AR45 KD experiments, human myoblasts were treated with 1 μM AR45-targeting siRNA (SBMA-1) or NTT for 48 hours in 50:50 skeletal muscle growth medium/Opti-MEM I–reduced serum with 5% (v/v) FBS. DHT (10 nM) was added to the cells 24 hours after transfection. siRNA sequences are available in fig. S5.

### Generation of RNA-seq libraries

RNA was extracted from flash-frozen patient samples, cells, or murine tissues homogenized in TRIzol-chloroform and purified using the Ambion PureLink RNA Mini Kit or Qiagen RNeasy Mini Kit (74104, QIAGEN). RNA integrity was assessed using the Bioanalyzer (G2939BA, Agilent).

For the human tissues, RNA-seq libraries were prepared from 500 ng of total RNA using the KAPA Stranded RNA-seq Kit with RiboErase (Kapa Biosystems, 07962304001) for ribosomal RNA depletion and Illumina compatible indexes (NEXTflex RNA-seq Barcodes, NOVA-512915, PerkinElmer). Pooled libraries (average insert size of 375 bp) were sequenced on an Illumina HiSeq 2500 platform using a paired-end 125 nucleotide setting to yield 40 million to 50 million reads per library. For the iPSC-derived motor neurons, RNA-seq libraries were prepared from 500 ng of total RNA using the RNA-Seq Stranded RiboZero Gold. Sequencing was paired-end (2 × 75 bp) for target depth of 100 million read pairs per sample using the Illumina HiSeq 4000 platform. Library preparation and sequencing were performed by the Bioinformatics Section, National Institute of Neurological Disorders and Stroke, National Institutes of Health (USA).

For the human myoblasts where AR45 was knocked down, mRNA libraries were generated using 1.5 μg of total RNA and the NEBNext Ultra II Directional RNA Library Prep Kit for Illumina (New England BioLabs, E7760). Libraries were multiplexed and pair-end sequenced on the Illumina HiSeq 4000 platform. For the murine TA muscles, RNA-seq polyA libraries were prepared from 900 ng of total RNA using the TruSeq Stranded mRNA kit (Illumina, 20020595) and pair-end sequenced (2 × 150 bp) on the Illumina NovaSeq 6000 system. Library preparation and sequencing were performed by the Oxford Genomics Centre, The Wellcome Centre for Human Genetics, University of Oxford, England.

### RNA-seq analysis pipeline

For the brain and iPSC dataset, paired-end sequence files (fastq) per sample were quality-inspected using the FastQC tool (www.bioinformatics.babraham.ac.uk/projects/fastqc/), then adaptor-clipped (TruSeq3-PE-2.fa:2:30:10), and trimmed to remove 5′ nucleotide bias (HEADCROP:12) and low quality calls (TRAILING:20 SLIDINGWINDOW:4:20 MINLEN:15) using the Trimmomatic tool (www.usadellab.org/cms/?page=trimmomatic). Surviving intact pairs of reads per sample were reference-mapped against the current instance of the human genome (GRCh38.82). Expression per known annotated gene (Homo_sapiens.GRCh38.82.chr.gtf) in transcripts per kilobase million units was pedestalled by 2 and then log_2_-transformed. Genes not having an expression value of >1 posttransformation for at least one sample were discarded as not detected, while expression across samples for genes not discarded was quantile normalized. To assure quality of the data postnormalization, exploratory inspection was performed using Tukey box plot, covariance-based principal components analysis (PCA) scatterplot, and correlation-based heatmap. To remove noise-biased expression values, locally weighted scatterplot smoothing (LOWESS) was applied across normalized expression for all genes by sample class (coefficient of variation ~ mean expression). LOWESS fits were then overplotted and inspected to identify the common low-end expression value where the relationship between mean expression (i.e., “signal”) and coefficient of variation (i.e., “noise”) grossly deviated from linearity. Expression values were then floored to equal this value if less, while expression for genes not observed greater than this value for at least one sample was discarded as noise-biased. For genes not discarded, expression differences across sample classes were tested for using the one-factor analysis of variance (ANOVA) test under Benjamini-Hochberg (BH) false discovery rate multiple comparison correction condition using sample class as the factor. Genes having a type III–corrected *P* < 0.05 by this test were then subset and the TukeyHSD post hoc test used to generate mean differences and *P* values for each possible pairwise comparison of classes. Genes having a post hoc *P* < 0.05 for a specific comparison and a linear difference of means ≥1.5× for the same comparison were deemed to have expression significantly different between the compared classes respectively. After testing, sample-to-sample relationships were investigated via covariance-based PCA scatterplot and Pearson correlation–based clustered heatmap using the unique union of genes deemed to have a significant difference of expression between at least two classes.

For the human myoblasts and murine tissues dataset, sequence reads were adapted and quality trimmed with Trim Galore! (v 0.4.1; https://github.com/FelixKrueger/TrimGalore). Quality control on both raw and trimmed reads was done with FastQC (v 0.11.7; www.bioinformatics.babraham.ac.uk/projects/fastqc/) and MultiQC (v 0.9) (*77*). For human myoblasts datasets, trimmed reads were then aligned to the human reference transcriptome (Gencode v29; www.gencodegenes.org/human/release_29.html) with Salmon (v 0.12) (*78*). The resulting quantification files were combined and read into R using the tximport package (v 1.12.3), and differential expression analysis was performed with DESeq2 (v 1.24) (*79*). For murine tissue datasets, trimmed reads were aligned to the mouse genome (Ensembl GRCm38) using HISAT2 (v 2.2.0) (*80*), alignment files were processed using SAMTools (v1.10) (*81*), and reads were counted in known genes (Mus_musculus.GRCm38.101.gtf) using the htseq-count function and –m union argument from HTSeq (v0.12.4) (*82*). Read counts files were combined using Python, and differential expression analysis was performed using DESeq2. Differences between groups were tested using the DESeq2 contrast function, and BH-adjusted *P* values were reported.

Pathway analysis was performed on differentially expressed genes using the GSEA package (v 1.10.0) (*83*) with gene set permutation. Unless otherwise stated, all analysis was performed using default parameters. Heatmap was generated using the Multiple Experiment Viewer (v 4.8.1) (*84*). For murine tissues datasets, normalized counts were used to generate heatmap with *k*-means clustering (15 clusters each for up-regulated and down-regulated genes in the SBMA AR100Q samples). Clusters that showed reversed disease gene expression upon AAV9.AR45 treatment were combined to give lists of genes that were partially corrected by AR45 overexpression.

Motif scanning tool, Find Individual Motif Occurrences, was used to investigate the presence of ARE motif on the gene promoters (*85*). ARE motif or AR binding motif was obtained from JASPAR database, MA0007.2 for human AR binding sites, and MA0007.3 for mouse AR binding sites. Gene promoter was defined as region −1000 to +100 bp from RefSeq TSS. ggsashimi plot was generated using the scripts publicly available on GitHub with default parameters (*86*).

### Cycloheximide chase assay

MCF7 cells were transfected with 1 μg of FLAG-AR45 plasmid at 40% confluency. Eight hours after transfection, cells were treated with 10 nM DHT or equal volume ethanol. After 12 hours, 100 μg of cycloheximide in DMSO (Sigma-Aldrich) was added to the cells (*t* = 0) and cells were harvested at 0 and 24 hours for protein expression analysis. Untransfected MCF7 cells with cycloheximide treatment were included for comparisons.

### Immunoblotting ELISA

Cells were lysed in 2× SDS–polyacrylamide gel electrophoresis (SDS-PAGE) buffer [4% (w/v) SDS, 100 mM tris-base, 20% (v/v) glycerol, and 0.008% (w/v) bromophenol blue] supplemented with PhosSTOP and cOmplete, mini, EDTA-free protease inhibitor cocktail (Roche) at 4°C. Lysates were boiled at 100°C for 10 min before centrifugation. Supernatants were collected, and protein levels were measured and adjusted by Pierce BCA Protein Assay (Thermo Fisher Scientific). For Western blot analysis, proteins were separated on NuPAGE 10% bis-tris gel (Invitrogen) and transferred onto polyvinylidene difluoride membranes using Invitrogen Novex XCell SureLock Mini-Cell and XCell II blot module. Membranes were incubated overnight with primary antibody in 1:1000 dilution, anti-AR antibody (Santa Cruz Biotechnology, H-280 or Abcam, ab52615), anti-tubulin (Thermo Fisher Scientific, 14-4502-82), or anti-vinculin (Sigma-Aldrich, hVIN-1) and, for 1 hour with secondary antibody in 1:10,000 dilution, anti–mouse-horseradish peroxidase (HRP)–conjugated antibody (Invitrogen, 62-6520) or anti–rabbit-HRP conjugated antibody (Invitrogen, 31460). Protein signals were detected in the presence of Pierce ECL Western blotting substrate (Thermo Fisher Scientific) using LI-COR Odyssey Infrared Imaging System. For cycloheximide chase experiments, quantifications were performed using the ImageStudio software (Li-Cor Biosciences).

For FLAG-AR45 co-IP experiments, cells were lysed with lysis buffer [50 mM tris-HCl, 0.1 M NaCl, 1% (v/v) NP-40, and 10% (v/v) glycerol (pH 7.5)] supplemented with protease inhibitor cocktail (Roche) at 4°C. Lysate was cleared by centrifugation, and the supernatant was precleared through incubation with protein G agarose beads (Invitrogen). The supernatant was then incubated overnight at 4°C with monoclonal anti-FLAG M2–conjugated magnetic beads (Sigma-Aldrich, M8823), or untagged beads (Chromotek bab-20), as a control. Beads were washed three times with wash buffer [0.1 M tris-HCl, 0.3 M NaCl, and 1% (v/v) Triton X-100 (pH 7.5)], which was followed by elution with 1× sample buffer, separation on SDS-PAGE gels, and analysis by Western blotting following standard protocols.

For AR co-IP experiments to assess AR interaction with BRD4, cells were lysed and nuclear fractions were obtained using NE-PER^TM^ nuclear and cytoplasmic extraction kit following the manufacturer’s protocol (Thermo Fisher Scientific). One microgram of mouse monoclonal anti-AR antibody (Santa Cruz Biotechnology, 441, sc-7305) or monoclonal anti-mouse IgG1 isotype control antibody (Cell Signaling Technology, 5415s) was coupled to protein G magnetic Dynabeads (Invitrogen). Nuclear extracts were incubated overnight at 4°C with the antibody-conjugated beads. Beads were washed for 10 times in 1% PBS with Tween 20, followed by elution in 2× SDS protein gel loading buffer at 100°C for 10 min and separation on SDS-PAGE gels and Western blotting.

Serum testosterone levels in AR45- versus mock-treated AR100Q mice at 11 weeks of age were measured using the Testosterone Parameter Assay Kit (R&D Systems, KGE010) according to the manufacturer’s protocol.

### Immunofluorescence and immunohistochemistry

Spinal cord and quadriceps muscle tissues were dissected and snap-frozen in dry ice/isopropanol slurry. Tissue sections (10 μm thick) were stained with H&E or NADH to ascertain the overall morphology and pathologic changes. For immunofluorescence experiments, cross sections of spinal cord, quadriceps muscle, TA muscle, and liver tissues were permeabilized in 0.1% Triton X-100 and blocked in PBS with 4% bovine serum albumin and 2% normal goat serum. Sections were incubated overnight at 4°C in 1:100 dilution with anti-NCAM (ProteinTech, 14255-1-AP), anti–P-NCAM (EMD Millipore, 5324), anti-ubiquitin (Abcam, ab7780), anti-laminin (Sigma-Aldrich, L0663), anti-ChAT (Novusbio, NBP1-30052), or anti-F4/80 (Abcam, ab131260) antibodies followed by incubation for 1 hour (20° to 25°C) with the appropriate Alexa Fluor–conjugated secondary antibodies (1:1000; Thermo Fisher Scientific). Sections were mounted with the VECTASHIELD Antifade Mountant with 4′,6-diamidino-2-phenylindole (DAPI) (Vector Laboratories) and allowed to dry for at least 24 hours at (20° to 25°C) before imaging. To determine the number of inclusions, tissue sections were stained with 1C2 antibody (1:5000; EMD Millipore, 5TF1-1C2) using the Mouse on Mouse Basic Kit (Vector Laboratories, BMK-2202). For the purposes quantification, whole slides containing six sections of tissue 50 μm apart from three different animals were imported into the 3D Histec Pannoramic 250 slide scanner (3D Histech, Hungary). This was performed at Bioimaging Facility, University of Manchester. Images from at least four contiguous sections were taken using the CaseViewer software and analyzed by a blinded investigator to the treatment using Fiji software (*87*).

To detect FL-AR45 protein via immunofluorescence, human myoblast cells grown on poly-l-lysine–coated cover slips, sections of spinal cord, and TA muscles of AR100Q mice were fixed in cooled methanol for 10 min at −20°C and then in cooled acetone for 1 min at −20°C. Sections or cells were incubated overnight at 4C in 1:80 dilution with rabbit anti-FLAG (Sigma-Aldrich, F7425) antibody followed by incubation for 1 hour at room temperature with secondary anti-rabbit IgG Alexa Fluor 555 antibody (Invitrogen, A21428) at 1:1000 dilution and DAPI staining for 3 min. Sections or cells were mounted with DAKO fluorescence mounting medium (DAKO, s3023) and imaged with the EVOS FL Auto Imaging System (Thermo Fisher Scientific) or the 3D Histec Pannoramic 250 slide scanner.

### Statistical analyses

Survival and time to disease onset of SBMA mice were determined by Kaplan-Meier estimation, and comparisons were made with the log-rank test. A two-way ANOVA was conducted to compare the effect of the treatment on weights, rotarod performances, and grip strength of the animals using treatment as a between-subjects factor and time as a within-subjects factor. All other data were analyzed by a two-tailed *t* test analysis. GraphPad Prism version 8 was used to perform the statistical analyses. Power analysis was performed using G*Power 3.1.9.2 software.

## SUPPLEMENTARY MATERIALS

Supplementary material for this article is available at http://advances.sciencemag.org/cgi/content/full/7/34/eabi6896/DC1

## Appendix

View/request a protocol for this paper from *Bio-protocol*.
